# A Qualitative Meta-Analysis Reveals Consistent Effects of Atrazine on Freshwater Fish and Amphibians

**DOI:** 10.1289/ehp.0901164

**Published:** 2009-09-23

**Authors:** Jason R. Rohr, Krista A. McCoy

**Affiliations:** Department of Integrative Biology, University of South Florida, Tampa, Florida, USA

**Keywords:** aromatase, behavior, disease, gonads, immunity, metamorphosis, parasite, reproduction, testicular ovarian follicles, vitellogenin

## Abstract

**Objective:**

The biological effects of the herbicide atrazine on freshwater vertebrates are highly controversial. In an effort to resolve the controversy, we conducted a qualitative meta-analysis on the effects of ecologically relevant atrazine concentrations on amphibian and fish survival, behavior, metamorphic traits, infections, and immune, endocrine, and reproductive systems.

**Data sources:**

We used published, peer-reviewed research and applied strict quality criteria for inclusion of studies in the meta-analysis.

**Data synthesis:**

We found little evidence that atrazine consistently caused direct mortality of fish or amphibians, but we found evidence that it can have indirect and sublethal effects. The relationship between atrazine concentration and timing of amphibian metamorphosis was regularly nonmonotonic, indicating that atrazine can both accelerate and delay metamorphosis. Atrazine reduced size at or near metamorphosis in 15 of 17 studies and 14 of 14 species. Atrazine elevated amphibian and fish activity in 12 of 13 studies, reduced antipredator behaviors in 6 of 7 studies, and reduced olfactory abilities for fish but not for amphibians. Atrazine was associated with a reduction in 33 of 43 immune function end points and with an increase in 13 of 16 infection end points. Atrazine altered at least one aspect of gonadal morphology in 7 of 10 studies and consistently affected gonadal function, altering spermatogenesis in 2 of 2 studies and sex hormone concentrations in 6 of 7 studies. Atrazine did not affect vitellogenin in 5 studies and increased aromatase in only 1 of 6 studies. Effects of atrazine on fish and amphibian reproductive success, sex ratios, gene frequencies, populations, and communities remain uncertain.

**Conclusions:**

Although there is much left to learn about the effects of atrazine, we identified several consistent effects of atrazine that must be weighed against any of its benefits and the costs and benefits of alternatives to atrazine use.

The herbicide atrazine (2-chloro-4-ethylamino- 6-isopropyl-amino-s-triazine) is the second most commonly used pesticide in the United States (Kiely et al. 2004) and perhaps the world (Solomon et al. 1996; van Dijk and Guicherit 1999). It is a photosynthesis inhibitor used to control certain annual broadleaf weeds, predominantly in corn but also in sorghum, sugarcane, and other crops and landscaping. The environmental risk posed by atrazine to aquatic systems is presently being reevaluated by the U.S. Environmental Protection Agency (U.S. EPA 2003, 2007). One of the challenges in evaluating the safety of atrazine has been that its biological effects are highly controversial, and much of the debate in the literature has been targeted at its effects on freshwater vertebrates (Hayes 2004; Renner 2004).

There have been four reviews on the biological effects of atrazine, all of which were funded by the corporation that produced or produces this chemical (Giddings et al. 2005; Huber 1993; Solomon et al. 1996, 2008). However, none of the past reviews used a meta-analytical approach to identify generalities in responses to atrazine exposure. Meta-analysis, as paraphrased from the U.S. EPA, is the systematic analysis of studies examining similar end points to draw general conclusions, develop support for hypotheses, and/or produce an estimate of overall effects (U.S. EPA 2009a). This sort of weight-of-evidence approach would provide directional hypotheses for future work on atrazine. Furthermore, it would offer invaluable information to regulatory agencies on general and expected impacts of atrazine on freshwater vertebrates that might help resolve much of the controversy surrounding atrazine. Given the lack of a meta-analytical assessment and the potential importance of any atrazine effects, we set out to conduct an objective, qualitative meta-analysis on the effects of atrazine on amphibian and fish survival, behavior, metamorphic traits, and immune, endocrine, and reproductive systems.

## Atrazine Persistence, Transport, and Exposure

To place the results of this meta-analysis within an ecologic context and to evaluate the relevance of studied atrazine concentrations and exposure regimes, we briefly discuss the fate, transport, and field concentrations of atrazine. Atrazine is persistent relative to most current-use pesticides. Ciba-Giegy Corporation (1994), the company that previously produced atrazine, reported no detectable change in atrazine concentration after 30 days in hydrolysis studies conducted at pHs between 5 and 7, and an aqueous photolysis half-life of 335 days under natural light and a neutral pH. Half-lives from field and mesocosm studies are variable because degradation can depend on various environmental conditions. Nevertheless, several field and mesocosm studies report half-lives > 3 months (e.g., de Noyelles et al. 1989; Klaassen and Kadoum 1979).

Atrazine is also relatively mobile—regularly entering water bodies through runoff—and concentrations in surface waters often peak after rains. Several researchers have suggested that atrazine can be transported 1,000 km aerially (van Dijk and Guicherit 1999). Indeed, atrazine has been found regularly in surface waters and precipitation great distances from where it is used, such as above the Arctic Circle, albeit at low concentrations (van Dijk and Guicherit 1999).

Wet deposition of atrazine might also be important in some areas. In a review on atmospheric dispersion of current-use pesticides, van Dijk and Guicherit (1999) reported more studies detecting atrazine in rain or air (from European and U.S. sites) than any other current-use pesticide. The maximum reported wet deposition of atrazine is 154 μg/L from Iowa precipitation (Hatfield et al. 1996). Wet deposition > 1 μg/L was reported regularly in North America and Europe between 1980 and the early 1990s (reviewed by van Dijk and Guicherit 1999). As a reference point, the maximum contaminant level for drinking water set by the U.S. EPA is 3 μg/L atrazine (U.S. EPA 2002).

Surface water is likely the primary source of atrazine exposure for freshwater vertebrates. Data on atrazine concentrations in surface water, however, are more abundant for lotic (streams and rivers) than lentic (lakes, ponds, wetlands, ditches) systems (Solomon et al. 2008), primarily because of the extensive stream monitoring conducted by the U.S. Geological Survey National Water Quality Assessment project and Syngenta Crop Protection, Inc. (U.S. EPA 2007). In lentic systems, water is not replenished as it is in lotic systems, and chemicals can concentrate as lentic systems dry. Maximum reported concentrations in lentic systems are often 2.5–10 times higher than maximum concentrations in lotic systems (Baker and Laflen 1979; Edwards et al. 1997; Evans and Duseja 1973; Frank et al. 1990; Kadoum and Mock 1978; Kolpin et al. 1997). Additionally, many amphibians develop in ephemeral agricultural ponds that might receive and concentrate atrazine (Knutson et al. 2004).

Given the limited data on atrazine concentrations in lentic systems, the expected (or estimated) environmental concentration (EEC) is a reasonable alternative for estimating concentrations to which aquatic organisms are likely to be exposed. GENEEC2 software (U.S. EPA 2009b) calculates standardized EECs used by the U.S. EPA for Tier-1 chemical risk screening. EECs are important because chemical registration decisions entail comparing lowest observable effect concentrations (LOECs) to EECs to determine whether higher-level modeling is warranted. Hence, effects of a chemical near or below the EEC can affect the decision to approve its use.

For present atrazine application rates, EECs based on GENEEC2 software are typically near 100 μg/L but can be higher for some crops. However, the recommended application rates (~ 2 lb active ingredient/acre) are now two to four times less than they were in the early 1990s (~ 8 lb active ingredient/acre). Hence, at the time of atrazine registration, LOECs near or below 500 μg/L, a feasible EEC at the time, might have triggered Tier-2 testing and might have raised concerns about the safety of atrazine that could have compromised its registration. Given both past and present-day conditions, the lack of thorough data on atrazine concentrations in lentic systems, and the common use of agricultural ponds, ditches, and wetlands by amphibians and fish, we suggest that concentrations near or below historical EECs (≤ 500 μg/L) are ecologically relevant when considering the findings of this meta-analysis. This is arguably conservative given that atrazine concentrations > 500 μg/L have been regularly recorded in agricultural ponds and ditches (Baker and Laflen 1979; Edwards et al. 1997; Evans and Duseja 1973; Frank et al. 1990; Kadoum and Mock 1978; Kolpin et al. 1997).

## Methods

We selected studies for this meta-analysis beginning with those cited by Solomon et al. (2008), the most recent review of atrazine effects on amphibians and fish. We then supplemented these studies by searching Web of Science (Thomson Reuters, New York, NY) to identify studies that might have been missed by Solomon et al. (2008). The search terms were “atrazine” combined with either “amphibian*” or “fish*”.

Selection criteria for inclusion of studies in meta-analyses can affect the conclusions that are drawn (Englund et al. 1999). Hence, we excluded from this meta-analysis studies that had substantial contamination in control treatments or reference sites (unless a regression approach was taken to analyze the data); no presentation of statistics and within-group variance estimates; considerable inconsistencies that could affect the biological conclusions; spatial confounders associated with atrazine treatments; pseudoreplication; or other considerable flaws in experimental design. We evaluated whether the exclusion of these studies changed the conclusion of the meta-analysis for each end point (Englund et al. 1999). For the 15 response variables, the inclusion of studies that did not meet our criteria never altered the conclusions of our meta-analyses, and in some cases including these studies actually strengthened the conclusions. Because of this and space limitations, studies that were excluded and why, as well as the directions of effects in these studies, are provided in Supplemental Material available online (doi:10.1289/ehp.0901164.S1 via http://dx.doi.org/).

To conduct a qualitative meta-analysis, we chose to use the vote-counting method—in which we tallied the number of studies that did and did not detect effects of atrazine—for several reasons. We quantified the effects of atrazine on 15 response variables from > 125 studies, and vote counting, the simplest approach to meta-analyses, made it feasible to manage this complexity. Vote counting also facilitates identifying response variables that might warrant more sophisticated meta- analyses based on effect sizes. Finally, we chose vote counting because it is a conservative approach, biasing results toward detecting no overall effect (Gurevitch and Hedges 1993). Because most atrazine studies conducted analysis of variance to test for dose responses, despite regression analyses providing much greater statistical power (Cottingham et al. 2005), we include studies that had substantial trends for effects of atrazine (i.e., a nonsignificant increase or decrease) with studies that reported statistically significant effects (α = 0.05). Our criteria for a trend were a clear dose response, a probability value < 0.1, or authors interpreting their nonsignificant result as a trend. Never did including trends change our conclusions of the meta-analysis.

## Results and Discussion

### Effects of atrazine on fish and amphibian survival

Many researchers have evaluated the effects of atrazine on fish (reviewed by Giddings et al. 2005; Huber 1993; Solomon et al. 1996) and amphibian survival (e.g., Allran and Karasov 2000, 2001; Brodeur et al. 2009; Diana et al. 2000; Freeman and Rayburn 2005; Rohr et al. 2003, 2004, 2006b). Our general conclusions from these studies are consistent with the conclusions of authors from previous atrazine reviews (Giddings et al. 2005; Huber 1993; Solomon et al. 1996, 2008): There is not consistent, published evidence that ecologically relevant concentrations of atrazine are directly toxic to fish or amphibians. There are, however, some important exceptions (e.g., Alvarez and Fuiman 2005; Rohr et al. 2006b, 2008c; Storrs and Kiesecker 2004). Because our conclusions are consistent with previous reviews, we did not conduct a meta-analysis on survival.

### Effects of atrazine on fish and amphibian development and growth. Background on metamorphosis

A basic understanding of four concepts about amphibian metamorphosis is necessary to interpret the effects of any chemical on time to, or size at, metamorphosis. First, amphibians must reach a minimum size before they can metamorphose (Wilbur and Collins 1973). Second, once they reach this size, they can accelerate development and metamorphose earlier if they are in a stressful environment or metamorphose later if they are in a good environment (Wilbur and Collins 1973). Last, metamorphosis is predominantly controlled by corticosterone and thyroid hormones (Larson et al. 1998); thus endocrine system disruption can lead to inappropriately timed metamorphosis.

These important facts have profound implications for understanding the effects of pollution on metamorphic traits. For example, imagine that an amphibian shunts energy away from growth to detoxify a chemical and, as a result, reaches the minimum size for metamorphosis 5 days later than amphibians not exposed to the chemical. Once this amphibian reaches the minimum size for metamorphosis, it might accelerate its developmental rate and metamorphose 5 days earlier to get out of the stressful chemical environment. In this example, there is no net effect of the chemical on time to metamorphosis despite inarguably having considerable effects on energy use, growth, and development (Larson et al. 1998). A single chemical could delay, accelerate, or have no effect on timing of metamorphosis, depending on chemical type and concentration.

This example highlights four points. First, a lack of an effect of a chemical on timing of metamorphosis does not mean there was no effect on developmental rate or hormones that drive metamorphosis, as concluded by Solomon et al. (2008). Second, nonmonotonic dose responses in the timing of metamorphosis are expected and are likely common. This is because there are several processes occurring (detoxification, growth, and modulation of developmental timing) that can be temporally offset and that likely have different (and potentially opposite) functional responses to the same chemical. Third, timing of metamorphosis in response to chemicals should be highly variable. This variation should not be interpreted as inconsistencies across studies (e.g., Solomon et al. 2008), because the complexity of metamorphosis is expected to induce extreme variability. Finally, unlike timing of metamorphosis, size at metamorphosis is expected to monotonically decrease with increasing chemical concentration across species and studies (controlling for time to metamorphosis) because energy used for detoxification is often taken away from that used for growth and development.

#### Effects on metamorphic traits

Our qualitative meta-analysis on the effects of atrazine on metamorphic traits is consistent with the predictions described above. Twelve of 21 studies found significant effects of atrazine on metamorphic timing, with 7 showing an increase and 7 showing a decrease in time to metamorphosis; thus, as predicted, the direction of the effect was not consistent across studies (Table 1). Seven of the 21 studies had either clear nonmonotonic dose responses or were possibly nonmonotonic (Table 1). These results are consistent with the high variability and high probability of nonmonotonicity expected for this end point.

Only two studies explicitly quantified the effects of atrazine on both thyroid hormones and timing of metamorphosis, and both showed significant nonmonotonic effects (Freeman et al. 2005; Larson et al. 1998) (Table 1). Further, Larson et al. (1998) revealed delays in growth and development early in life followed by accelerated development and early metamorphosis once a critical size for metamorphosis was reached. Additional studies that quantify the impacts of atrazine on thyroid hormones, corticosteroid hormones, and changes in growth and development through time are needed.

In contrast to timing of metamorphosis, size at metamorphosis shows a clear dose-dependent response to atrazine exposure (Table 1). Fifteen of 17 studies and 14 of 14 species showed significant reductions, or considerable trends toward reductions, in amphibian size at metamorphosis associated with atrazine exposure, and all of these studies reported effects at ecologically relevant concentrations based on the above criteria (Table 1). Similar growth reductions have been observed in fish (Alvarez and Fuiman 2005; McCarthy and Fuiman 2008). Atrazine consistently reduced amphibian size, which is likely to have adverse effects on amphibian populations because smaller metamorphs generally have lower terrestrial survival, lower lifetime reproduction, and compromised immune function (Carey et al. 1999; Scott 1994; Smith 1987). However, population-level effects of atrazine have not been empirically tested for in nature and thus need to be evaluated explicitly.

### Effects of atrazine on fish and amphibian behavior

Effects on locomotor activity. Twelve of 13 studies reported that atrazine exposure increased amphibian or fish locomotor activity over at least a portion of the concentration gradient tested (Table 2). Interestingly, 4 of 5 studies on fish, but none of the studies on amphibians, reported nonmonotonic dose responses. For fish, low concentrations of atrazine stimulated hyperactivity, but higher concentrations caused reductions in activity. For amphibians, hyperactivity was typically observed at the concentrations tested, but higher concentrations would likely eventually become toxic and reduce activity. All studies conducted on fish detected effects of atrazine on locomotor activity, whereas 88% of the studies on amphibians detected atrazine effects (Table 2).

The effects of atrazine on amphibian and fish locomotor activity are consistent with atrazine-induced changes in locomotor activity in mammals. Atrazine seems to cause hyperactivity in mammals by competing with receptors for the inhibitory neurotransmitter gamma-aminobutyric acid, by altering monoamine turnover, and through neurotoxicity of the dopaminergic system (Das et al. 2001; Rodriguez et al. 2005). One study showed that atrazine has similar effects on the nervous system of Ranid frogs (Papaefthimiou et al. 2003), but additional studies are needed that evaluate the mechanisms responsible for atrazine-induced activity changes in fish and amphibians.

#### Effects on antipredator behaviors

Six of 7 studies reported that atrazine decreased amphibian and fish behaviors associated with predation-related risk reduction (Table 2). Reduced predation avoidance behaviors can increase predation risk, whereas increased hyperactivity should increase encounter rates with predators (Skelly 1994). Hence, reduced risk-reduction behaviors coupled with hyperactivity are expected to increase predation. However, there are no published studies on the effects of atrazine on predator–prey relationships of which we are aware. Given that atrazine might have effects on both predators and prey, the effects of atrazine on predator–prey interactions are difficult to predict without additional studies.

#### Effects on olfaction

Five of 5 studies reported that atrazine exposure reduced olfactory sensitivity of fish in a dose-dependent manner (Table 2). In contrast, 3 of 3 studies on amphibians detected no effects of atrazine on olfaction at much higher concentrations than were tested on fish (Table 2). One study on amphibians stained activated olfactory neurons with agmatine and found no difference in the stimulation of olfactory neurons between atrazine-treated and control animals (Lanzel 2008).

#### Effects on other behaviors

One study showed that atrazine reduced amphibian water-conserving behaviors, which increased their rate of water loss (Rohr and Palmer 2005) (Table 2). Interestingly, both the hyperactivity and the reduced water-conserving behaviors occurred hundreds of days after atrazine exposure had ceased; there was no evidence that these end points recovered from atrazine exposure, suggesting permanent effects (Rohr and Palmer 2005). Amphibians are extremely susceptible to desiccation; thus atrazine-induced changes in water conserving behaviors would be expected to increase mortality risk.

### Effects of atrazine on fish and amphibian immunity and infections

Effects on immunity. Our qualitative meta-analysis revealed that atrazine exposure consistently reduced immune functioning of fish and amphibians, with 16 of 18 studies finding effects at ecologically relevant concentrations. However, many of the end points (16 of 39) were from studies where atrazine was tested as part of a mixture of pesticides, and thus the effects of atrazine were not isolated (Table 3). Nevertheless, atrazine exposure—alone (21 of 27 end points) or in a pesticide mixture (12 of 16 end points)—was associated with reduced immune functioning, resulting in an overall reduction in 77% (33 of 43) of the quantified fish and amphibian immune end points (including trends for a decrease) (Table 3). These results are somewhat conservative because in one study multiple genes associated with immunity were significantly down-regulated (Langerveld et al. 2009), but they were counted as a single end point (Table 3).

#### Effects on infections

Similar to the effects of atrazine on amphibian and fish immunity, atrazine exposure was consistently associated with an increase in infection end points in fish and amphibians at ecologically relevant concentrations (Table 4). Atrazine elevated trematode, nematode, viral, and bacterial infections (Table 4). Of the studies with sufficient statistical power and without obvious confounders, 12 of 14 of the infection end points increased or showed a strong trend toward increasing, indicating either more infected individuals, more infections per individual, faster maturation, or greater reproduction of the parasite within the host, or greater parasite-induced host mortality (Table 4). As with immunity, these patterns should be considered with caution because many of these end points (6 of 16) came from studies where atrazine was part of a mixture of pesticides tested. Nevertheless, atrazine exposure, alone (4 of 7 end points) or in a pesticide mixture or field study (9 of 9 end points), was associated with an increase in infection end points (Table 4). In general, high concentrations of atrazine seem to be directly toxic to trematodes and viruses, possibly reducing infection risk for amphibians (Forson and Storfer 2006a; Koprivnikar et al. 2006; Rohr et al. 2008b), whereas more ecologically common concentrations seem to increase amphibian susceptibility, elevating infection risk (Forson and Storfer 2006b; Gendron et al. 2003; Kiesecker 2002; Rohr et al. 2008c).

Several atrazine studies collected immunologic data only from animals that were also exposed to parasites, thus confounding immune parameters with parasite exposure and loads (Christin et al. 2003; Forson and Storfer 2006b; Gendron et al. 2003; Hayes et al. 2006; Kiesecker 2002; Rohr et al. 2008c). However, in each of these studies, atrazine was associated with both reduced immune parameters and elevated parasite loads. The elevated infections associated with atrazine cannot be explained by parasites reducing immune responses. Hence, the parsimonious explanation for both of these findings is that atrazine reduced immune responses, which elevated infections, especially given that it is often beneficial for vertebrates to up-regulate immunity upon infection (Raffel et al. 2006).

Despite the apparent consistency in the effects of atrazine on immunity and infections (Table 3), much remains to be learned about the effects of atrazine and other chemicals on parasite–host interactions (Raffel et al. 2008; Rohr et al. 2006a). For instance, we know little about how atrazine-induced changes affect population or community dynamics or most human diseases.

### Effects of atrazine on fish and amphibian gonadal morphology

#### General morphologic end points

Sex differentiation is the process by which gonads develop into either testes or ovaries from an undifferentiated or bipotential gonad (Hayes 1998). This process is distinct from reproductive maturation where the differentiated gonad becomes reproductively functional (e.g., undergoes spermatogenesis in males). Determining if atrazine induces changes in gonadal morphology is an important step in evaluating whether it can influence sexual differentiation.

Atrazine consistently affected male gonadal morphology in fish and amphibians (Table 5). Seven of the 10 studies including results on males and females reported strong trends or statistically significant alterations (6 studies) in at least one aspect of general gonadal morphology associated with atrazine exposure. Alterations included discontinuous and multiple testes, sexually ambiguous gonadal tissue, testicular ovarian follicles (TOFs), altered gonadal somatic index (GSI; ratio of gonad weight to body weight), expanded testicular lobules, and spermatogenic tubule diameter (Table 5).

Effects on ovarian morphology are generally less obvious than those on testicular morphology and are typically dismissed without quantification. None of the three studies on fish or amphibians included in our meta-analysis found significant effects of atrazine on ovarian morphology, suggesting that atrazine induces fewer gonadal abnormalities in females than males. However, additional studies are necessary to fully evaluate the effects of atrazine on female gonadal morphology.

#### TOFs as a natural phenomenon

Jooste et al. (2005) and Solomon et al. (2008) argued that experiments with high numbers of TOFs in control *Xenopus laevis* support the hypothesis that TOFs are normal in some *X. laevis* populations. Although it was argued long ago that some anurans in some environments transition through a hermaphroditic phase during development (Witschi 1929), the literature we reviewed does not argue that adult amphibians commonly have oocytes within testicular tissue or are naturally hermaphroditic (Eggert 2004; Hayes 1998). Indeed, *X. laevis* sexually differentiates (without a transitional/hermaphroditic stage) during the larval period prior to sexual maturation (Iwasawa and Yamaguchi 1984). Thus, cases of gonadal abnormalities in healthy adult *X. laevis* populations should be rare. Given that simultaneous hermaphroditism has not been previously reported in *X. laevis* despite decades of research on their reproductive biology, an equally or more plausible explanation for high numbers of TOFs in control animals (e.g., Jooste et al. 2005; Orton et al. 2006) is exposure to some type of unmeasured endocrine-disrupting contaminant.

### Effects of atrazine on fish and amphibian sex ratios

Given that atrazine exposure has been proposed to feminize gonadal development (Hayes et al. 2002, 2003), it might lead to female-biased sex ratios. Many studies, however, have severe methodologic errors, such as contaminated controls or inadequate data reporting [see Supplemental Material, Table S1 (doi:10.1289/ehp.0901164.S1)], preventing a conclusive synthesis of the effects of atrazine on sex ratios. None of the sex-ratio studies used the most accepted and powerful approaches for testing for changes in sex ratios (e.g., Wilson and Hardy 2002). Only four studies, all on *X. laevis*, were of sufficient quality to be included in our meta-analysis, and only one found that atrazine induced a female-biased sex ratio (see Supplemental Material, Table S2 (doi:10.1289/ehp.0901164.S1)].

### Effects of atrazine on fish and amphibian gonadal function

Chemicals that alter gonadal development can affect gonadal function, such as germ cell (e.g., spermatogenesis in males) and steroid hormone production (McCoy et al. 2008; McCoy and Guillette, in press), and thus can lead to altered reproductive success.

#### Effects on testicular cell types

Spermatogenesis is the process through which mature male gametes (spermatozoa) are produced from precursor cells (spermatogenic cells). The relative ratios of different spermatogenic cell types, rather than abundance of spermatozoa alone, is the most sensitive metric of altered spermatogenesis. Unfortunately, few studies on effects of atrazine on spermatogenesis met our inclusion criteria. Two of two studies demonstrated that atrazine was associated with altered spermatogenesis and that several cell types were affected (Table 6). Thus, atrazine appears capable of altering spermatogenesis, but the contexts and generality of these effects cannot be firmly established. Our analysis once again highlights a need for more rigorous investigations.

#### Effects on sex hormone concentrations

Sex hormone production is an important function of gonads that can be altered by gonadal abnormalities (McCoy et al. 2008). Indeed, altered hormone concentrations are the defining characteristic, in many cases, of endocrine disruption. Six of seven studies on fish and amphibians document strong trends or significantly (five studies) altered sex hormone concentrations associated with atrazine exposure (Table 6). Although many of these studies were conducted in the field and are therefore correlative, the consistency of these results across studies suggests that atrazine alters sex hormone production and should be considered an endocrine-disrupting chemical. A more thorough understanding of the effects of atrazine on hormone concentrations will require more detailed studies that account for the inherent variability of endocrine system processes.

#### Effects on reproductive success

Reproductive success is strongly linked to population persistence and is likely one of the most important end points in toxicologic studies. Five studies that evaluated the effects of atrazine on measures of reproductive success met our meta-analysis requirements (Table 6). Two studies on adult fish, *Pimephales promelas*, found no significant effect of atrazine on number of eggs produced, fertilization success, proportion of hatchlings, or larval development. However, one of these studies (Bringolf et al. 2004) found several nonsignificant, adverse trends (Table 6). Two of three studies on amphibians found no effects of atrazine on hatching success, whereas one showed reduced hatching success and delayed hatching (Table 6). Given the mixed results, the effect of atrazine on reproductive success needs to be studied more thoroughly.

### Effects of atrazine on fish and amphibian vitellogenin

Vitellogenin is an egg yolk precursor protein produced in the livers of female fish and amphibians. Estrogens induce vitellogenin synthesis in both males and females *in vivo*, and quantification of vitellogenin is now an accepted screening test for estrogenic effects of chemicals (Scholz and Mayer 2008). None of the five studies (four on fish) found significant effects of atrazine on circulating or whole-body concentrations of vitellogenin [see Supplemental Material, Table S2 (doi:10.1289/ehp.0901164.S1)]. Hence, these data do not support the hypothesis that atrazine is strongly estrogenic to fish.

### Effects of atrazine on fish and amphibian aromatase

Cytochrome p450 aromatase catalyzes the conversion of androgens to estrogens in gonads and is critical for maintaining a balance between these sex hormone classes. Hayes et al. (2002) hypothesized that decreases in testosterone associated with atrazine exposure in their study could be driven by an atrazine-induced increase in aromatase and a concomitant increase in the conversion of testosterone and other androgens to estrogens. This hypothesis seemed reasonable because atrazine was known to increase aromatase in human cancer cell lines and in alligator gonadal–adrenal mesonephros (Crain et al. 1997; Sanderson et al. 2000). However, since 2002, several studies have explicitly tested whether atrazine increases aromatase in fish and amphibians, and only one of six studies included in our meta-analysis found that atrazine was associated with increased aromatase gene expression [see Supplemental Material, Table S2 (doi:10.1289/ehp.0901164.S1)].

### Effects of atrazine on fish and amphibian populations and communities

Although there are too few studies examining the effects of atrazine on freshwater vertebrate populations to warrant meta-analysis, and virtually all community-level studies infer—rather than test for—indirect effects (Rohr and Crumrine 2005), the effects of atrazine on populations and communities warrants a brief discussion. Any chemical that affects physiology, growth, development, reproduction, survival, or species interactions can affect population and community dynamics (Clements and Rohr 2009; Rohr et al. 2006a). However, the effects of contaminants might not result in immediate population declines because the survivors of chemical exposure frequently have less competition for resources, thus providing density-mediated compensation for adverse effects of the chemical (Rohr et al. 2006b). Demonstrating that a factor is the cause of any population decline is, indeed, incredibly difficult (Rohr et al. 2008a). Rohr et al. (2006b) revealed significant and delayed declines in *Ambystoma barbouri* salamander populations at 4, 40, and 400 μg/L atrazine, above and beyond the counteracting effects of density-mediated compensation. Although this study provided greater ecologic realism than many studies on atrazine, caution should be taken extrapolating these effects to populations in nature because this study was conducted in laboratory terraria. There is certainly a need for controlled studies on the effects of pesticides on wildlife populations.

Several studies have examined the effects of atrazine on amphibian and fish communities (Boone and James 2003; de Noyelles et al. 1989; Kettle 1982; Rohr and Crumrine 2005; Rohr et al. 2008c). Many of these studies reported alterations in fish or amphibian growth and abundance that seem to be caused by atrazine-induced changes in photosynthetic organisms (reviewed by Giddings et al. 2005; Solomon et al. 2008). At ecologically relevant concentrations, atrazine is expected to have a bevy of indirect effects by altering the abundance of periphyton, phytoplankton, and macrophytes (Huber 1993; Solomon et al. 1996). However, none of these studies distinguish between direct and indirect effects of atrazine on fish or amphibians.

There are several field studies comparing amphibian populations or species richness between atrazine-exposed and unexposed habitats (Bonin et al. 1997; Du Preez et al. 2005; Knutson et al. 2004). All of these studies are correlational, and none thoroughly considered or ruled out alternative hypotheses for the observed patterns.

### Caveats

We would be remiss not to mention some caveats regarding this meta-analysis. First, a problem with many meta-analyses is the “file-drawer” effect. This refers to the fact that researchers tend to place the results of experiments showing no effects in their file drawer, and many journals tend to publish fewer studies showing no effects than those with effects (Gurevitch and Hedges 1993; Osenberg et al. 1999). This might be less of a problem in studies on pesticides because these chemicals are designed to kill biota; thus in many cases, the null hypothesis might be an effect rather than the absence of one. Additionally, a substantial industry contingent works to ensure that both significant and nonsignificant effects of chemicals get published. Indeed, in the review of atrazine by Solomon et al. (2008), there were approximately 63 cases where atrazine had significant adverse effects and 70 cases where atrazine had no significant effects (Rohr JR, McCoy KA, unpublished data), suggesting that the file-drawer effect is unlikely to be strongly biasing submission and publication of nonsignificant atrazine results. However, we cannot completely discount the possibility that the file-drawer effect generated a bias toward greater publication of significant effects of atrazine.

Another admonishment is that some of the end points in this meta-analysis were not independent of one another. For example, we tallied multiple end points from a single study despite the possibility that they might not be entirely independent.

Finally, we must consider the findings of this meta-analysis on atrazine relative to alternative strategies for weed control. If the alternative to atrazine is another chemical, then we should ideally compare the effects of atrazine to the replacement chemical. In fact, atrazine might be less detrimental to freshwater vertebrates than a replacement herbicide. If the alternative to atrazine does not entail a chemical replacement, then the effects revealed here might indeed be disconcerting. However, we also cannot ignore the benefit, if any, that atrazine provides. Interestingly, several studies estimate that atrazine increases corn yields by only 1–3% (reviewed by Ackerman 2007). To adequately evaluate any chemical, we should ideally conduct a thorough cost– benefit analysis that considers the focal chemical and alternatives to its use and is based on comprehensive and accurate knowledge [see Ackerman (2007) for a review and critique of atrazine cost–benefit analyses].

## Conclusions

As in past reviews, we found little evidence that atrazine consistently causes direct mortality of freshwater vertebrates at ecologically relevant concentrations, but there is evidence that atrazine might have adverse indirect ecologic effects. However, in contrast to a previous review on atrazine (Solomon et al. 2008), we unveiled consistent effects of atrazine at ecologically relevant concentrations for many other response variables in our meta-analysis. The discrepancy between our findings and the conclusions of previous reviews could be partly a function of differences in criteria for including studies in the group used to draw general conclusions about atrazine effects. Past reviews (e.g., Solomon et al. 2008) did not clearly define their inclusion criteria, did not make it clear which studies affected their conclusions (or how they came to their conclusions), and regularly dismissed significant effects of atrazine.

Here we reveal that, for freshwater vertebrates, atrazine consistently reduced growth rates, had variable effects on timing of metamorphosis that were often nonmonotonic, elevated locomotor activity, and reduced antipredator behaviors. Amphibian and fish immunity was reliably reduced by ecologically relevant concentrations of atrazine, and this was regularly accompanied by elevated infections. Atrazine exposure induced diverse morphologic gonadal abnormalities in fish and amphibians and was associated with altered gonadal function, such as modified sex hormone production. This suggests that atrazine should be considered an endocrine-disrupting chemical. Finally, we do not have a thorough appreciation of the reproductive repercussions of atrazine.

Several end points had enough well-conducted studies to warrant more sophisticated meta-analyses based on effect sizes (e.g., growth, timing of metamorphosis, activity, immunity, infections, gonadal abnormalities). Meta-analyses based on effect sizes can provide parameter and standard errors estimates and thus can be useful for probabilistic risk assessment and for predicting atrazine effects.

Although we found consistent effects of atrazine on freshwater vertebrates, the consequences of these effects remain uncertain. We know little about how atrazine-induced changes in vertebrate growth, somatic development, behavior, immunity, gonadal development, or physiology affect reproduction, populations, gene frequencies, or communities. However, it was Sir Austin Bradford Hill who wisely stated in his address to the Royal Society of Medicine in 1965 that

All scientific work is incomplete [and] . . . liable to be upset or modified by advancing knowledge. That does not confer upon us freedom to ignore the knowledge we already have, or to postpone action that it appears to demand at a given time. (Hill 1965)

Whatever action is taken in the re-evaluation of atrazine by the U.S EPA, we strongly encourage regulators to consider the consistent effects of atrazine on various taxa and to weigh these effects against any benefits atrazine provides and the alternatives to atrazine use.

## Correction

Corrections have been made from the original manuscript published online: Criteria for identifying results showing “substantial trends” has been clarified; the number of studies has been corrected in the text; and the “effect direction” for relevant studies has been corrected in Tables 1, 3, and 5.

## Figures and Tables

**Table 1:** Summary of the results for the effects of atrazine on the developmental rate and size at or near metamorphosis for amphibians.

	Net effect on developmental rate				Size at or near metamorphosis								
Taxon, species	Effect direction	Conc where effect was observed (μg/L)	Nonmono-tonic dose response	Excluded from meta-analysis?	Effect direction	Conc where effect was observed (μg/L)	Nonmono-tonic dose response	Excluded from meta-analysis?	Conc tested (μg/L)	Atrazine grade	Experiment type	Exposure duration	Reference
Frog													
*Bufo americanus*	ND	–	NA	No	↓	200	NA	No	200	Comm; Aatrex^a^	PE	≤ 88 days	Boone and James 2003^b^
*B. americanus*	↓^c^	250, 500, 1,000	Yes	No	↓^d^	No Conc differed from controls	No	No	250, 500, 1,000, 5,000, 10,000	Tech	SR	3 weeks	Freeman et al. 2005
*B. americanus*	ND	–	No	No	No data	–	No data	Yes	1, 3, 30	Tech	SR	LTM	Storrs and Semlitsch 2008
*Rhinella arenarum*	↑ at 100 and 1,000, ↓ at 5,000	100, 1,000, 5,000	Yes	No	No data	–	No data	Yes	100, 1,000, 5,000	Tech	SR	LTM	Brodeur et al. 2009
*Hyla chrysoscelis*	↑	192	No	No	No data	–	No data	Yes	96, 192	Tech	PE, two pulses	≤ 129 days	Briston and Threlkeld 1998^b^
*Hyla versicolor*	ND^e^	–	Possibly	No	↓	200, 2,000	No	No	20, 200, 2,000	Tech	PE	Mean of 13 days	Diana et al. 2000^f^
*H. versicolor*	ND	–	NA	No	No data	–	No data	Yes	1, 3, 30	Tech	SR	LTM	Storrs and Semlitsch 2008
*Rana clamitans*	↓	10	Yes	No	↓	10	Yes	No	10, 25	Tech	SR	≤ 273 days	Coady et al. 2004^f^
*Rana pipiens*	Unknown^g^	–	No	Yes	↓^h^	Not tested	No	No	20, 200	Tech	SR	LTM	Allran and Karasov 2000
*R. pipiens*	ND	–	NA	No	↓	0.1	NA	No	0.1	Tech	SR	LTM	Hayes et al. 2006
*R. pipiens*	ND	–	NA	No	ND	–	NA	No	5	Not provided	SR	ETM, ≤ 45 days	Bridges et al. 2004^i^
*Rana sphenocephala*	ND	–	NA	No	↓	200	NA	No	200	Comm; Aatrex^a^	PE	≤ 57 days	Boone and James 2003^b^
*R. sphenocephala*	ND	–	NA	No	No data	–	No data	Yes	1, 3, 30	Tech	SR	LTM	Storrs and Semlitsch 2008
*Rana sylvatica*	No data	–	No data	Yes	↓	Unknown; conc in ponds not provided	NA	No	3, 30	Comm	FS	Unknown	Kiesecker 2002^j^
*Xenopus laevis*	No data	–	No data	Yes	ND	–	No	No	1, 10, 25	Tech	SR	Mean of 56 days	Carr et al. 2003
*X. laevis*	ND	–	NA	No	No data	–	No data	Yes	1, 10, 25	Tech	SR	ETM	Du Preez et al. 2008
*X. laevis*	↑	100, 450, 800	No	No	Unknown^k^	–	Unknown	Yes	100, 450, 800	Tech	SR	4 weeks	Freeman and Rayburn 2005
*X. laevis*	Unknown l,m,n	–	Unkown	Yes	↓^o^	0.01, 1, 100	Possibly	No	0.01, 0.1, 1.0, 25, and 100	Tech	SR	≤ 75 days	Kloas et al. 2009
*X. laevis*	↓ detected by regression	No Conc differed from controls	No	No	↓	20, 40, 80, 160, 320	No	No	20, 40, 80, 160, 320	Tech	SR	LTM	Sullivan and Spence 2003
*X. laevis*	No data	–	NA	Yes	↓	400	NA	No	400	Tech	SR	LTM	Langerveld et al. 2009

Salamander													
*Ambystoma barbouri*	↑	40, 400	No	No	↓	400	No	No	4, 40, 400	Tech	SR	Mean of 52 days exposure	Rohr et al. 2004
*Ambystoma macrodactylum*	↑	184	No	No	↓	184	No	No	1.84, 18.4, 184	Tech	SR	30 days	Forson and Storfer 2006a
*Ambystoma tigrinum*	↑	16 vs. 1.6, but not vs. 0	Possibly; no data	No	ND; trend toward ↓^p^	–	No data	No	1.6, 16, 160	Tech	SR	LTM	Forson and Storfer 2006b
*Ambystoma maculatum*	↑ and ↓^q^	250	Yes	No	↓	250	No	No	75, 250	Tech	SR	86 days	Larson et al. 1998
*A. maculatum*	↓	200	NA	No	↓	200	NA	No	200	Comm; Aatrex^a^	PE	≤ 57 days	Boone and James 2003^e^
*Ambystoma texanum*	↓	200	NA	No	↓	200	NA	No	200	Comm; Aatrex^a^	PE	≤ 88 days	Boone and James 2003^b^,^r^

Abbreviations: ↓, decreased; ↑, increased; Comm, commercial; Conc, concentration; ETM, embryo to metamorphosis, or earlier (cases where amphibians metamorphosed before atrazine exposure ceased); FS, field survey; LTM, early larvae to metamorphosis; NA, not applicable (used when there were too few concentrations to evaluate nonmonotonicity); ND, not detected; PE, pulse experiment; SR, static renewal experiment; Tech, technical. Excluded studies are listed in Supplemental Material, Table S1 (doi:10.1289/ehp.0901164.S1).

a Aatrex is 59.2% inactive ingredients.

b Community-level study.

c Authors show that atrazine modifies the thyroid axis for both *X. laevis* and *B. americanus.*

d All five atrazine concentrations tested reduced frog size relative to controls, but no within-group variance estimates were provided.

e 200 ppb developed faster than 2,000 ppb.

f Only a single egg mass; might not reflect general response.

g Use only 50% of the metamorphs in the time to metamorphosis analysis without describing how they selected this subset of metamorphs or why they used only 50% for time to metamorphosis but 100% of the metamorphs for size at metamorphosis.

h Authors report an interaction between atrazine and time for frog length, indicating that control animals were larger than those exposed to atrazine by the end of the experiment.

i Tested as a mixture of 5 μ/L atrazine and 5 μ/L carbaryl.

j Compared ponds with and without atrazine; effects might be due to other factors.

k Frogs lose weight at metamorphosis, thus mass measurements were confounded by grouping tadpole and metamorph weights.

l Provide no within-group variance estimate.

m No statistics provided but conclude that there was no effect of atrazine.

n Graphs for developmental rate through time are indiscernible.

o Detected effects in only one of two experiments and for females only.

p *p* = 0.080 for regression analysis, one-tailed test.

q Results depended on developmental stage; authors showed that atrazine modifies thyroxine and corticosterone hormones.

r Results depended on drying conditions.

**Table 2:** Summary of the results for the effects of atrazine on fish and amphibian behaviors.

Taxon, species	End point	Effect direction	Conc where effect was observed (μg/L)	Conc tested (μg/L)	Nonmonotonic dose response	Atrazine grade	Experiment type	Exposure duration	Reference
Locomotor activity									
Salamander									
*A. barbouri*	Locomotor activity after disturbance	↑	400	4, 40, 400	No	Tech	SR	37 days	Rohr et al. 2003
*A. barbouri*	Locomotor activity after disturbance	↑	400	4, 40, 400	No	Tech	SR	Mean of 52 days; LTM	Rohr et al. 2004
*A. barbouri*	Locomotor activity after disturbance	↑	40, 400	4, 40, 400	No	Tech	SR	Mean of 47 days; LTM	Rohr and Palmer 2005
*A. barbouri*	Locomotor activity	↑	400	40, 400, 800	No	Tech	PE	4 days	Rohr et al. (unpublished data)

Frog									
*R. sylvatica*	Locomotor activity	↑	Two doses of 25 separated by 2 weeks	Two doses of 25 separated by 2 weeks	NA	Tech	PE	1 month	Rohr and Crumrine 2005^a^
*B. americanus*	Locomotor activity	ND	–	201	NA	Tech	PE	4 days	Rohr et al. 2009
*X. laevis*	Abnormal swimming	↑	25	1, 10, 25	No	Tech	SR	Mean of 56 days, LTM	Carr et al. 2003
*H. chrysoscelis*	Burst swimming	↑	Positive dose response	96, 192	No	Tech	PE, two pulses	≤ 129 days, LTM	Briston and Threlkeld 1998

Fish									
*Carassius auratus*	Burst swimming	↑	0.5, 50	0.5, 5, 50	Possibly	Tech	PE	1 day	Saglio and Tijasse 1998
*C. auratus*	Burst swimming	↑	0.1, 1, 10	0.1, 1, 10	Possibly	Tech	PE	1 day	Saglio and Tijasse 1998
*Oncorhynchus mykiss*	Locomotor activity	↑	1, 10	1, 10, 100	Yes	Tech	PE	30 min	Tierney et al. 2007
*Lepomis cyanellus*	Locomotor activity	↑/↓	400 but not 800	40, 400, 800	Yes, only in presence of natural prey	Tech	PE	4 days	Rohr et al. (unpublished data)
Larval *Sciaenops ocellatus*^b^	Locomotor activity and abnormal swimming	↑	40, 80	40, 80	No	Tech	PE	72 hr	Alvarez and Fuiman 2005

Predation-related risk reduction									
Salamander									
*A. barbouri*	Refuge use	↓, detected with regression	None	4, 40, 400	No	Tech	SR	37 days	Rohr et al. 2003
*A. barbouri*	Refuge use	↓	400	4, 40, 400	No	Tech	SR	Mean of 52 days, LTM	Rohr et al. 2004

Frog									
*R. sylvatica*	Refuge use	↓	Two doses of 25 separated by 2 weeks	Two doses of 25 separated by 2 weeks	NA	Tech	PE, two pulses	1 month	Rohr and Crumrine 2005^a^
*C. auratus*	Grouping	↓	5, 50	0.5, 5, 50	No	Tech	PE	1 day	Saglio and Tijasse 1998
*C. auratus*	Sheltering in presence of predator cue	↓	5	0.5, 5, 50	Possibly	Tech	PE	1 day	Saglio and Tijasse 1998
*C. auratus*	Grouping in presence of predator cue	↓	5	0.5, 5, 50	Possibly	Tech	PE	1 day	Saglio and Tijasse 1998
Larval *S. ocellatus*^b^	Predation rates	ND	40, 80	40, 80	No	Tech	PE	72 hr	Alvarez and Fuiman 2005

Olfaction									
Frog									
*B. americanus*	Chemical detection of food, parasites, and predator cues	ND	–	201	NA	Tech	PE	4 days	Rohr et al. 2009
Salamander									
*Plethodon shermani*	Chemical detection of food or sex pheromones	ND	–	300	NA	Tech	SR	28 days	Lanzel 2008
*P. shermani*	Activated olfactory neurons	ND	–	700	NA	Tech	SR	28 days	Lanzel 2008

Fish									
*Salmo salar*	Olfactory response (electroolfactogram)	↓	2, 5, 10, 20	0.1, 1, 2, 5, 10, 20	No	Tech	PE	30 min	Moore and Waring 1998
*S. salar*	Olfactory response (electroolfactogram)	↓	1	0.5, 1	No	Tech	PE	30 min	Moore and Lower 2001
*S. salar*	Olfactory response (electroolfactogram)	↓	0.5, 1	0.5, 1	No	Tech	PE	30 min	Moore and Lower 2001^c^
*O. mykiss*	Olfactory response (electroolfactogram)	↓	10, 100	1, 10, 100	No	Tech	PE	30 min	Tierney et al. 2007
*O. mykiss*	Response ratio to l-histidine	↓	10	1, 10, 100	Possibly	Tech	PE	30 min	Tierney et al. 2007

Other behaviors									
Salamander									
*A. barbouri*	Water-conserving behaviors	↓	40, 400	4, 40, 400	No	Tech	SR	Mean of 52 days; LTM	Rohr and Palmer 2005^d^

Abbreviations: ↓, decreased; ↑, increased; Conc, concentration; LTM, early larvae to metamorphosis; NA, not applicable (used when there were too few concentrations to evaluate nonmonotonicity); ND, none detected; conc, concentration; tech, technical; PE, pulse experiment; SR, static renewal experiment; Tech, technical. Excluded studies are listed Supplemental Material, Table S1 (doi:10.1289/ehp.0901164.S1).

a Community-level study.

b Larval red drum are often found in freshwater, so they were included in this meta-analysis.

c Mixture of 0.5:0.5 and 1.0:1.0 atrazine and simazine; thus, total concentration of triazine was 1 and 2 ppb, respectively.

d Increased salamander water loss and thus desiccation risk.

**Table 3:** Summary of the results for the effects of atrazine, through water column exposure, on fish and amphibian immunity.

Taxon, species	End point	Effect direction	Conc where effect was observed (μg/L)	Conc tested (μg/L)	Nonmonotonic dose response^a^	Atrazine grade	Experiment type^b^	Exposure duration	Reference
Salamander									
*A. tigrinum*	No. of peripheral leukocytes	↓	16, 160	1.6, 16, 160	No	Tech	SR	Until metamorphosis	Forson and Storfer 2006b

Frog									
*R. pipiens*	Splenocyte viability	ND	–	2.1, 21, 210	No	Tech	SR	21 days	Christin et al. 2003, 2004a
*R. pipiens*	No. of splenocytes	↓, if using appropriate one-tailed test	210	2.1, 21, 210	No	Tech	SR	21 days	Christin et al. 2003, 2004a
*R. pipiens*	No. of phagocytic splenocytes	↓ postinfection	210	2.1, 21, 210	No	Tech	SR	21 days	Christin et al. 2003^a^
*R. pipiens*	T cell proliferation	↓ in presence of mitogens	2.1, 21, 210	2.1, 21, 210	No	Tech	SR	21 days	Christin et al. 2003, 2004a
*R. pipiens*	T cell proliferation	↓ in absence of mitogens	2.1, 21, 210	2.1, 21, 210	No	Tech	SR	21 days	Christin et al. 2003, 2004a
*R. pipiens*	Absolute no. of phagocytic cells in spleen	↓	2.1, 21, 210	2.1, 21, 210	No	Tech	SR	21 days	Christin et al. 2004^a^
*R. pipiens*	No. of thymic plaques	↑, indicating reduced immune capacity^b^	0.1	0.1	NA	Tech	SR	Until metamorphosis	Hayes et al. 2006
*R. pipiens*	No. of hemolytic plaques representing antibody secreting B cells	↓	1, 10	1, 10	No	Not provided	SR	4 weeks	Houck and Sessions 2006
*R. pipiens*	No. of lymphocyte from spleen	ND	–	1, 10	Possibly	Not provided	SR	8 weeks	Houck and Sessions 2006
*R. pipiens*	No. of white blood cells	↓	0.01 to 10	0.01, 0.1, 1, 10	No	Tech	SR	8 days	Brodkin et al. 2007^c^
*R. pipiens*	No. of highly phagocytic cells	↓	0.01 to 10	0.01, 0.1, 1, 10	No	Tech	SR	8 days	Brodkin et al. 2007^c^
*X. laevis*	Splenocyte viability	ND	–	2.1, 21, 210, 2,100	No	Tech	SR	21 days	Christin et al. 2004^a^
*X. laevis*	Splenocyte cellularity	↓	210, 2100	2.1, 21, 210, 2,100	No	Tech	SR	21 days	Christin et al. 2004^a^
*X. laevis*	Relative no. of phagocytic cells in spleen	↑	21, 210, 2,100	2.1, 21, 210, 2,100	No	Tech	SR	21 days	Christin et al. 2004^a^
*X. laevis*	Absolute no. of phagocytic cells in spleen	↓	210, 2,100	2.1, 21, 210, 2,100	No	Tech	SR	21 days	Christin et al. 2004^a^
*X. laevis*	T cell proliferation	ND	–	2.1, 21, 210, 2,100	No data	Tech	SR	21 days	Christin et al. 2003^a^
*X. laevis*	Downregulation of several genes involved in skin peptide defense	↓	400	400	NA	Tech	SR	Until metamorphosis	Langerveld et al. 2009
*X. laevis*	Downregulation of several genes involved in blood cell function	↓	400	400	NA	Tech	SR	Until metamorphosis	Langerveld et al. 2009
*R. sylvatica*	No. of eosinophil from circulating blood	↓	3, 30	3, 30	No	Tech	SR	4 weeks	Kiesecker 2002
*R. pipiens*	No. of melano-macrophages from liver	↓	< 1 Do not know maximum concentration	Unknown	No	Comm	FS	Unknown	Rohr et al. 2008c^d^
*Rana paulustris*	No. of melano-macrophages from liver	↓	117	117	NA	Tech	PE	4 weeks	Rohr et al. 2008c
*R. paulustris*	No. of eosinophil from liver	ND, trend toward decrease; *p =* 0.10	117	117	NA	Tech	PE	4 weeks	Rohr et al. 2008c
*R. clamitans*	No. of eosinophil from liver	↓	117	117	NA	Tech	PE	4 weeks	Rohr et al. 2008c
*R. clamitans*	No. of melano-macrophages from liver	ND	117	117	NA	Tech	PE	4 weeks	Rohr et al. 2008c

Fish									
*C. auratus*	No. of superoxide radical from macrophages of spleen and kidney	↑ 4 and 8 weeks; indicator of oxidative stress	42	42	NA	Tech	SR	12 weeks	Fatima et al. 2007^a^
*C. auratus*	Plasma lysozyme activity	↑ at 8 and 12 weeks, argued as a reduction in resistance to infection	42	42	NA	Tech	SR	12 weeks	Fatima et al. 2007^a^
*C. auratus*	Antibody titers against *Aeromonas hydrophila*	↓	42	42	NA	Tech	SR	12 weeks	Fatima et al. 2007^a^
*C. auratus*	Antioxidant enzyme in spleen (superoxide dismutase)	↓ at 4, 8, and 12 weeks	42	42	NA	Tech	SR	12 weeks	Fatima et al. 2007^a^
*Galaxias maculatus*	Leucocrit	↓	3, 50	0.9, 3, 10, 50	Possibly	Tech	SR	10 days	Davies et al. 1994
*O. mykiss*	Proliferative ability of circulating T lymphocytes (ConA)	↓	> 5,000	1,000–10,000	Possibly	Tech	PE	2 days	Rymuszka et al. 2007
*O. mykiss*	Proliferative ability of circulating B lymphocytes (LPS)	↓	> 5,000	1,000–10,000	Possibly	Tech	PE	2 days	Rymuszka et al. 2007
*O. mykiss*	Respiratory burst activity of circulating phagocytes	↓	> 2,500	1,000–10,000	Possibly	Tech	PE	2 days	Rymuszka et al. 2007
*Liza ramada* and *Liza aurata*	Macrophage quality	↓ (cells degenerated)	25–280	Unknown	Unknown	Unknown	Unknown	Unknown	Biagianti-Risbourg 1990^e^
*L. ramada* and *L. aurata*	Melanomacrophage centers in liver	↑	25–280	Unknown	Unknown	Unknown	Unknown	Unknown	Biagianti-Risbourg 1990^e^
*Salmonidae* (species not specified)	White blood cells	↓	100–1,000	Unknown	Unknown	Unknown	Unknown	Unknown	Walsh and Ribelin 1975^e^
*Salmonidae* (species not specified)	Lymphoid organ quality	↓ (evidence of atrophy)	100–1,000	Unknown	Unknown	Unknown	Unknown	Unknown	Walsh and Ribelin 1975^e^
*Salvelinus namaycush, Oncorhynchus kisutch*	Spleen weight	↓/no effect	1,500–13,500	Unknown	Unknown	Unknown	Unknown	Unknown	Zeeman and Brindley 1981
*S. namaycush, O. kisutch*	No. of lymphocytes	↓/no effect	1,500–13,500	Unknown	Unknown	Unknown	Unknown	Unknown	Zeeman and Brindley 1981

Abbreviations: ↓, decreased; ↑, increased; Comm, commercial; Conc, concentration; FS, field survey; NA, not applicable (used when there were too few concentrations to evaluate nonmonotonicity); ND, not detected; PE, pulse experiment; SR, static renewal experiment, Tech, technical. Excluded studies are listed in Supplemental Material, Table S1 (doi:10.1289/ehp.0901164.S1).

a Atrazine was a component of a mixture of pesticides tested, and thus the experiment did not isolate the effects of atrazine.

b Atrazine alone and every mixture containing atrazine increased thymic plaques.

c Immune response stimulated by thioglycollate.

d No quantified factors correlated with atrazine could parsimoniously explain patterns in infection.

e As reported by Dunier and Swicki 1993; could not obtain original works.

**Table 4:** Summary of the results for the effects of atrazine, through water column exposure, on fish and amphibian parasite infections.

Taxon, species	End point	Effect direction	Conc where effect was observed (μg/L)	Conc tested (μg/L)	Nonmonotonic dose response	Atrazine grade	Experiment type	Exposure duration	Reference
Salamander									
*A. macrodactylum*	Infectivity of ATV	↓	Not provided	1.84, 18.4, 184	Dose response not provided	Tech	SR	30 days	Forson and Storfer 2006a^a^
*A. tigrinum*	Percentage infected with ATV	↑ at 16 but not 1.6 or 160	16	1.6, 16, 160	Yes	Tech	SR	Until metamorphosis	Forson and Storfer 2006b^b^
*A. tigrinum*	Viral load	ND; *p* = 0.14	–	20, 200	No	Tech	SR	2 weeks	Kerby and Storfer 2009
*A. tigrinum*	Mortality due to ATV	↑	Not provided	20, 200	No	Tech	SR	2 weeks	Kerby and Storfer 2009

Frog									
*R. pipiens*	*Rhabdias ranae* nematode prevalence	ND; trend toward ↑	–	2.1, 21, 210	No	Tech	SR	21 days	Christin et al. 2003^c^
*R. pipiens*	No. of adult *R. ranae* nematode	↑, clear dose response	21 + 210 > controls, 210 > water control	2.1, 21, 210	No	Tech	SR	21 days	Gendron et al. 2003^c^
*R. pipiens*	*Chryseobacterium* (Flavobacterium) *menigosepticum* infections	↑	0.1	0.1	NA	Tech	SR	Until metamorphosis	Hayes et al. 2006^c^,^d^
*R. pipiens*	*R. ranae* nematode within host migration	Faster	21, 210	2.1, 21, 210	No	Tech	SR	21 days	Gendron et al. 2003^c^
*R. pipiens*	*R. ranae* nematode maturation and reproduction	Earlier	21, 210	2.1, 21, 210	No	Tech	SR	21 days	Gendron et al. 2003^c^
*R. sylvatica*	No. of *Ribieoria* sp. and *Telorchis* sp.	↑	3, 30	3, 30	No	Tech	SR	4 weeks	Kiesecker 2002
*R. sylvatica*	Limb deformities caused by *Ribieoria* sp.	↑ in ponds with atrazine	Ponds with atrazine	Unknown	NA	Comm	FS	Unknown	Kiesecker 2002
*R. clamitans*	No. of *Echinostoma trivolvis* cercariae	↑	201	201	NA	Tech	SR	2 weeks	Rohr et al. 2008b^e^
*R. pipiens*	No. of larval trematodes	↑	< 1 Do not know maximum Conc	Unknown	No	Comm	FS	Unknown	Rohr et al. 2008c^f^
*R. clamitans*	No. of larval *Plagiorchid* trematodes	↑	117	117	NA	Tech	PE	4 weeks	Rohr et al. 2008c
*R. clamitans*	No. of *Echinostoma trivolvis* cercariae	↓, but amphibians not exposed to atrazine	20, 200	20, 200	No	Comm; Aatrex^g^	PE	Cercariae exposed for 2 hr	Koprivnikar et al. 2006^h^,^i^,^j^

Fish									
*C. auratus*	Mortality due to *Aeromonas hydrophila* challenge	↑	42	42	NA	Tech	SR	12 weeks	Fatima et al. 2007^c^

Abbreviations: ↓, decreased; ↑, increased; ATV, *Ambystoma tigrinum* virus; Comm, commercial; Conc, concentration; FS, field survey; NA, not applicable (used when there were too few concentrations to evaluate nonmonotonicity); ND, not detected; PE, pulse experiment; SR, static renewal experiment, Tech, technical. Excluded studies are listed in Supplemental Material, Table S1 (doi:10.1289/ehp.0901164.S1).

a Effect was observed when combining of 1.84, 18.4, and 184 treatments and comparing with controls; effect might be predominantly due to 184.

b 160 ppb was thought to reduce ATV infectivity explaining nonmonotonicity.

c Atrazine was a component of a mixture of pesticides tested, and thus the experiment did not isolate the effects of atrazine.

d Saw this effect only when atrazine was mixed with eight other pesticides.

e Effect was found pooling pesticides and comparing them with control treatments.

f No quantified factors correlated with atrazine could parsimoniously explain patterns in infection.

g Aatrex is 59.2% inactive ingredients.

h Effects could be due to inactive ingredients.

i Effects could be due to chemicals other than atrazine that might be in the pond water used to make the stock solutions.

j All LC_50_s were calculated incorrectly.

**Table 5:** Summary of the effects of atrazine on general gonadal morphology.

Taxon, species	End point	Effect direction	Conc where effect was observed (μg/L)	Conc tested (μg/L)	Atrazine grade	Experiment type	Exposure duration	Reference
Testes								
Fish								
*Pimephales promelas*	Testis size corrected for body size	ND	5, 50	5, 50	Tech	SR	21 days	Bringolf et al. 2004^a^
*P. promelas*	Spermatogenic tubule diameter	↓	250	25, 250	Tech	FT	21 days	U.S. EPA 2005

Frog								
*X. laevis*	Discontinuous gonads (abnormal segmentation)	↑	25	1.0, 10, 25	Tech	SR	~78 days during larval period	Carr et al. 2003
*X. laevis*	Ambiguous gonads (not obviously male or female)	↑	25	1.0, 10, 25	Tech	SR	~78 days during larval period	Carr et al. 2003^b^
*X. laevis*	Testis size corrected for body size	↑	10	10, 100	Tech	SR	48 days	Hecker et al. 2005a^a^
*X. laevis*	Sperm/area	ND	–	10, 100	Tech	SR	48 days	Hecker et al. 2005a^a^
*X. laevis*	Testis size corrected for body size	ND	–	1, 25, 250	Tech	SR	36 days	Hecker et al. 2005a^a^
*R. clamitans*	Testis size corrected for body size	↓ in juvenile males	ND–3.13	ND–3.13^c^	Comm	FS	Unknown	McDaniel et al. 2008^c^
*R. pipiens*	TOFs (testicular oocytes)	↑ where atrazine was detected in 2003^c^	ND–3.14	ND–3.13^c^	Comm	FS	Unknown	McDaniel et al. 2008^c^,^d^
Various spp., mostly *R. clamitans*	Discontinuous testes (abnormal segmentation)	ND	–	ND–2^e^	Comm	FS	Unknown	Murphy et al. 2006a
Various spp., mostly *R. clamitans*	Intersex (having testicular and ovarian tissues)	ND	–	ND–2^e^	Comm	FS	Unknown	Murphy et al. 2006a
Various spp., mostly *R. clamitans*	TOFs (testicular oocytes)	↑ in 1 of 2 years in juveniles, positively correlated with max atrazine Conc in that year	ND–0.73	ND–2^e^	Comm	FS	Unknown	Murphy et al. 2006a
*R. clamitans*	Testis size corrected for body size	↑ in adult males at agricultural sites in 1 of 2 years	ND–250	ND–2^e^	Comm	FS	Unknown	Murphy et al. 2006b^f^
*X. laevis*	Hermaphroditism (testicular oocytes, intersex, mixed sex)	ND	–	0.1, 1, 10, 100	Tech	SR	~ 65 days during larval period	Oka et al. 2008
*Acris crepitans*	Intersex or testicular oocytes	Trend for ↑ *p* = 0.07	Atrazine detections	ND–70	Comm	FS	Unknown	Reeder et al. 1998^g^

Ovaries								
Fish								
*P. promelas*	Ovary size corrected for body size	Trend for ↓	50	5, 50	Tech	SR	21 days	Bringolf et al. 2004^a^
*P. promelas*	Proportion of oocytes undergoing atresia	ND	–	25, 250	Tech	FT	21 days	U.S. EPA 2005

Frog								
*H. versicolor, R. sphenocephala*	Ovarian developmental stage	ND	–	1, 3, 30^h^	Tech	SR	Through metamorphosis	Storrs and Semlitsch 2008
*B. americanus*	Ovarian developmental rate	ND	–	1, 3, 30^h^	Tech	SR	Through metamorphosis	Storrs and Semlitsch 2008

Abbreviations: ↓, decreased; ↑, increased; Comm, commercial; Conc, concentration; FS, field survey; FT, flow-through experiment; ND, not detected; SR, static renewal experiment, Tech, technical. Excluded studies are listed in Supplemental Material, Table S1 (doi:10.1289/ehp.0901164.S1).

a No test statistics or degrees of freedom are presented; however, means and variances were presented either in the text or in a figure of the article.

b *Xenopu*s are typically sexually differentiated at the gross morphologic level at metamorphosis; individuals in this study exposed to 25 μg/L were so sexually ambiguous they were initially considered intersex (having both testicular and ovarian issues).

c Atrazine concentration for the nonagricultural reference site during 2003 was reported incorrectly; repeated attempts to contact the authors for clarification have not been forthcoming.

d When atrazine concentrations were highest (2003), TOFs per individual occurred in higher numbers; TOFs were positively associated with atrazine, nitrate, and quantity of pesticides in a multivariate comparison, suggesting that atrazine is contributing to TOFs.

e Concentrations were between ND and 2 except on two occasions at one site, when levels were 65 and 250 μg/L.

f Authors argued that differences in GSI between agricultural and nonagricultural sites cannot be due to atrazine because GSI does not correlate with atrazine concentration; however, they presented no statistics to support this claim.

g The relationship between detection of atrazine and the presence of one or more intersex cricket frogs approached significance (*p* = 0.07).

h The actual concentration of the 30-μg/L treatment was 125 μg/L.

**Table 6:** Summary of the effects of atrazine on gonadal function.

Taxon, species	End point	Effect direction	Conc where effect was observed (μg/L)	Conc tested (μg/L)	Atrazine grade	Experiment type	Exposure duration	Reference
Testicular cell types								
Frog								
*R. clamitans*	Proportion of juvenile males with > 50% tubules containing spermatids and spermatozoa	Lower at agricultural site with highest atrazine concentrations	Range of medians, 0.068–0.78	ND–3.13^a^	Comm	FS	Unknown	McDaniel et al. 2008^a^
*R. pipiens*	Proportion of juvenile males with > 50% tubules containing spermatids and spermatozoa	Higher at agricultural site with highest atrazine concentrations	0.342 (mean of median concentrations)	ND–3.13^a^	Comm	FS	Unknown	McDaniel et al. 2008^a^

Fish								
*P. promelas*	Proportion of primary spermatogonia	↑	25, 250	25, 250	Test	FT	21 days	U.S. EPA 2005
*P. promelas*	Proportion of secondary spermatogonia	Reduced	25, 250	25, 250	Test	FT	21 days	U.S. EPA 2005

Sex hormone concentrations								
Frog								
*X. laevis*	Testosterone in adult males	↓	25	25	Tech	SR	46 days	Hayes et al. 2002^b^
*X. laevis*	Testosterone in adult males	ND	–	10, 100	Tech	SR	48 days	Hecker et al. 2005a
*X. laevis*	Estradiol in adult males	ND	–	10, 100	Tech	SR	48 days	Hecker et al. 2005a
*X. laevis*	Estradiol in adult males	ND	–	1, 25, 250	Tech	SR	36 days	Hecker et al. 2005b
*X. laevis*	Testosterone in adult males	↓	250	1, 25, 250	Tech	SR	36 days	Hecker et al. 2005b
*X. laevis*	Testosterone in females	↓ at agricultural sites, negatively correlated with concentration of atrazine and breakdown product	< 0.1–4.14	< 0.1–4.14	Comm	FS	Unknown	Hecker et al. 2004
*X. laevis*	Testosterone in males	Negatively correlated with diamino-chlorotriazine concentration (a product of atrazine breakdown)	< 0.1–4.14	< 0.1–4.14	Comm	FS	Unknown	Hecker et al. 2004
*X. laevis*	Estradiol in females	↓ at agricultural sites, negatively correlated with conc of atrazine and breakdown product	< 0.1–4.14	< 0.1–4.14	Comm	FS	Unknown	Hecker et al. 2004
*R. pipiens*	Testosterone in juvenile males (2003)	↓ at agricultural sites	Range of medians, 0.380–0.780	ND–3.13^a^	Comm	FS	Unknown	McDaniel et al. 2008^a^
*R. pipiens*	Testosterone in juvenile males (2003)	Negatively correlated with atrazine concentration	ND–3.13	ND–3.13^a^	Comm	FS	Unknown	McDaniel et al. 2008^a^,^c^
*R. pipiens*	11-Ketotestosterone in juvenile males (2003)	Negatively correlated with atrazine concentration	ND–3.13	ND–3.13^a^	Comm	FS	Unknown	McDaniel et al. 2008^a^,^c^
*R. pipiens*	Testosterone in adult females (2003)	Negatively correlated with atrazine concentration	ND–3.13	ND–3.13^a^	Comm	FS	Unknown	McDaniel et al. 2008^a^,^c^
*R. clamitans*	11-Ketotestosterone to testosterone ratio in adult females (late summer Aug–Sep 2002)	↑ at agricultural sites	Agricultural sites ranged from ND to 250	ND–250	Comm	FS	Unknown	Murphy et al. 2006b^d^
*R. clamitans*	11-Ketotestosterone to testosterone ratio in adult males (late summer Aug–Sep 2002)	↑ at agricultural sites	Agricultural sites ranged from ND to 250	ND–250	Comm	FS	Unknown	Murphy et al. 2006b^d^
*R. clamitans*	11-Ketotestosterone to testosterone ratio in adult males (early summer May 2003)	↑ at agricultural sites	Agricultural sites ranged from ND to 0.73	ND–250	Comm	FS	Unknown	Murphy et al. 2006b^d^
*R. clamitans*	Estradiol to testosterone ratio in adult females (late summer Aug–Sep 2002)	↑ at agricultural sites	Agricultural sites ranged from ND to 250	ND–250	Comm	FS	Unknown	Murphy et al. 2006b^d^
*R. clamitans*	Estradiol to testosterone ratio in adult males (Late summer Aug–Sep 2002)	↑ at agricultural sites	Agricultural sites ranged from ND to 250	ND–250	Comm	FS	Unknown	Murphy et al. 2006b^d^
*R. clamitans*	Estradiol to testosterone ratio in adult males (early summer May 2003)	↓ at agricultural sites	Agricultural sites ranged from ND to 0.73	ND–250	Comm	FS	Unknown	Murphy et al. 2006b^d^
*R. clamitans*	Estradiol to testosterone ratio in juvenile males (Jul 2003)	↑ at agricultural sites	Agricultural sites ranged from ND to 0.73	ND–250	Comm	FS	Unknown	Murphy et al. 2006b^d^
*R. clamitans*	Testosterone in adult males (early summer May 2003)	↑ at agricultural sites	Agricultural sites ranged from ND to 0.73	ND–250	Comm	FS	Unknown	Murphy et al. 2006b^d^
*R. clamitans*	Testosterone in juvenile females (Jul 2003)	↑ at agricultural sites	Agricultural sites ranged from ND to 0.73	ND–250	Comm	FS	Unknown	Murphy et al. 2006b^d^
*R. clamitans*	Testosterone in juvenile males (Jul 2003)	↑ at agricultural sites^d^	Agricultural sites ranged from ND to 0.73	ND–250	Comm	FS	Unknown	Murphy et al. 2006b^d^

Fish								
*P. promelas*	Testosterone female	ND	–	25, 250	Tech	FT	21 days	U.S. EPA 2005
*P. promelas*	Estradiol female	Trend (up to a 44% ↓)	25, 250	25, 250	Tech	FT	21 days	U.S. EPA 2005^e^
*P. promelas*	Testosterone male	Trend (up to a 31% ↓)	25, 250	25, 250	Tech	FT	21 days	U.S. EPA 2005^e^
*P. promelas*	11-Ketotestosterone male	Trend (up to a 47% ↓)	25, 250	25, 250	Tech	FT	21 days	U.S. EPA 2005^e^

Reproductive success								
Salamander								
*A. barbouri*	Proportion hatched and timing of hatching	ND	–	4, 40, 400	Tech	SR	37 days	Rohr et al. 2003
*A. barbouri*	Proportion hatched and timing of hatching	↓ and delayed hatching	400	4, 40, 400	Tech	SR	Mean of 52 days	Rohr et al. 2004

Frog								
*R. pipiens*	Proportion hatched	ND	–	2,590–20,000	Tech	SR	10 days	Allran and Karasov 2001
*R. clamitans*	Proportion hatched	ND	–	2,590–20,001	Tech	SR	10 days	Allran and Karasov 2001
*B. americanus*	Proportion hatched	ND	–	2,590–20,002	Tech	SR	10 days	Allran and Karasov 2001

Fish								
*P. promelas*	Eggs per spawning of exposed adults	Trend for a ↓	5	5, 50	Tech	SR	21 days	Bringolf et al. 2004^b^
*P. promelas*	Number of spawnings of exposed adults	Trend for a ↓	50	5, 50	Tech	SR	21 days	Bringolf et al. 2004^b^
*P. promelas*	Fertilization success of exposed adults	Trend for a ↓	50	5, 50	Tech	SR	21 days	Bringolf et al. 2004^b^
*P. promelas*	Proportion hatched and larval development of offspring from exposed adults	ND	–	5, 50	Tech	SR	21 days	Bringolf et al. 2004^b^
*P. promelas*	Egg production of exposed adults	ND	–	25, 250	Tech	FT	21 days	U.S. EPA 2005
*P. promelas*	Fertilization success of exposed adults	ND	–	25, 250	Tech	FT	21 days	U.S. EPA 2005
*P. promelas*	Proportion hatched and larval development of offspring from exposed adults	ND	–	25, 250	Tech	FT	21 days	U.S. EPA 2005

Abbreviations: ↓, decreased; ↑, increased; Comm, commercial; Conc, concentration; FS, field survey; FT, flow-through experiment; ND, not detected; SR, static renewal experiment, Tech, technical. Excluded studies are listed in Supplemental Material, Table S1 (doi:10.1289/ehp.0901164.S1).

a Atrazine concentration for the nonagricultural reference site during 2003 was reported incorrectly; repeated attempts to contact the authors for clarification have not been forthcoming.

b No test statistics or degrees of freedom were presented; however, means and variances were presented either in the text or in a figure of the article.

c Authors reported no significant correlation between atrazine and sex hormones in their abstract when, in fact, these end points were negatively correlated; contrary to the authors’ conclusion, the negative correlations across sexes and age groups reported in their study are unlikely to occur because of a low sample size or sampling error.

d Authors argued that differences in hormone levels between agricultural and nonagricultural sites cannot be due to atrazine because hormone concentrations do not correlate with atrazine concentration; however, they presented no statistics to support this claim.

e Low samples sizes (7–8 fish) likely precluded detecting these considerable effects.
