# Errata

**Published:** 2010-11

In the review by Rohr and McCoy [ Environ Health Perspect 118:20–32 (2010)], Orton et al. (2006) was cited on page 26 but was not included in the reference list. The reference is as follows:

Orton F, Carr JA, Handy RD. 2006. Effects of nitrate and atrazine on larval development and sexual differentiation in the northern leopard frog *Rana pipiens*. Environ Toxicol Chem 25:65–71.

